# Targeting the DNA replication stress phenotype of KRAS mutant cancer cells

**DOI:** 10.1038/s41598-021-83142-y

**Published:** 2021-02-11

**Authors:** Tara Al Zubaidi, O. H. Fiete Gehrisch, Marie-Michelle Genois, Qi Liu, Shan Lu, Jong Kung, Yunhe Xie, Jan Schuemann, Hsiao-Ming Lu, Aaron N. Hata, Lee Zou, Kerstin Borgmann, Henning Willers

**Affiliations:** 1 Department of Radiation Oncology, Massachusetts General Hospital, Harvard Medical School, 55 Fruit Street, Boston, MA 02114 USA; 2 Laboratory of Radiobiology and Experimental Radiooncology, Clinic of Radiotherapy and Radiooncology, University Medical Center Hamburg-Eppendorf, Hamburg, Germany; 3 Center for Cancer Research, Massachusetts General Hospital, Harvard Medical School, Charlestown, MA USA; 4 Shenzhen Bay Laboratory, Shenzhen, China; 5 Harbin Medical University Cancer Hospital, Harbin, China

**Keywords:** Cancer, Oncogenes

## Abstract

Mutant KRAS is a common tumor driver and frequently confers resistance to anti-cancer treatments such as radiation. DNA replication stress in these tumors may constitute a therapeutic liability but is poorly understood. Here, using single-molecule DNA fiber analysis, we first characterized baseline replication stress in a panel of unperturbed isogenic and non-isogenic cancer cell lines. Correlating with the observed enhanced replication stress we found increased levels of cytosolic double-stranded DNA in KRAS mutant compared to wild-type cells. Yet, despite this phenotype replication stress-inducing agents failed to selectively impact KRAS mutant cells, which were protected by CHK1. Similarly, most exogenous stressors studied did not differentially augment cytosolic DNA accumulation in KRAS mutant compared to wild-type cells. However, we found that proton radiation was able to slow fork progression and preferentially induce fork stalling in KRAS mutant cells. Proton treatment also partly reversed the radioresistance associated with mutant KRAS. The cellular effects of protons in the presence of KRAS mutation clearly contrasted that of other drugs affecting replication, highlighting the unique nature of the underlying DNA damage caused by protons. Taken together, our findings provide insight into the replication stress response associated with mutated KRAS, which may ultimately yield novel therapeutic opportunities.

## Introduction

The *KRAS* (Kirsten rat sarcoma 2 viral oncogene homolog) gene encodes a GTPase that is involved in signal transduction from the cell membrane to the nucleus^1,2^. The protein is most commonly mutated at codons 12 and 13, which causes constitutive activation of downstream signaling pathways and confers oncogenic properties. The *KRAS* oncogene is among the most prevalent tumor drivers, present in approximately 30% of non-small cell lung carcinoma (NSCLC), 40% of colorectal cancer, and 95% of pancreatic adenocarcinoma^1^. KRAS mutant (mut) cancers often exhibit poor drug responses and prognosis^3–8^. For the past two decades, it has been known that mutant KRAS also promotes cellular resistance to ionizing radiation^9–11^. However, only recently data from us and others have established that at least a subset of KRASmut cancers exhibit radioresistance in vivo and in cancer patients^12–17^. Strategies to overcome KRASmut radioresistance are being explored^18^.

There has been considerable effort devoted to identifying unique vulnerabilities of KRASmut tumors, in addition to more recent successes in directly targeting the protein^19,20^. Oncogenic KRAS induces DNA replication stress by promoting aberrations in the number of active replicons and replication fork progression, which leads to DNA damage and genomic instability^19^. As a result, cells respond by activating the DNA damage response. During this response stressed cells may become reliant on ATR and CHK1 kinases as well as RAD51 to promote continued proliferation in the presence of DNA damage^21–24^. Furthermore, the combined inhibition of WEE1 and PARP1, which presumably induces replication stress, was found to sensitize KRASmut tumor cells to ionizing radiation in vitro and *in vivo*^25^. However, there is remarkably little data analyzing the replication stress response in KRASmut cells using the single-molecule DNA fiber assay, a powerful method to investigate DNA replication fork processes^23,26–28^.

Under physiological conditions the cytoplasm of eukaryotic cells is virtually devoid of genomic DNA but several scenarios exist in which single-stranded (ss) and double-stranded (ds) DNA molecules are released into the cytosol from where cGAS-STING-dependent innate immune responses can be triggered^29^. In cancer cells, high levels of chromosomal instability were reported to maintain a cytosolic dsDNA pool leading to metastasis through non-canonical NF-κB signaling^30^. Another source of cytosolic dsDNA are mitochondria that are dysfunctional in the presence of LKB1 mutation^31^. DNA replication stress due to impaired DNA repair factors may also lead to export of DNA into the cytosol^32^, but how replication stress in oncogene-driven cancers affects cytosolic DNA production is poorly understood. Lastly, ionizing radiation is a potent inducer of cytosolic DNA in a dose-dependent manner, thereby mediating radiation-driven tumor rejection^33^.

Proton radiation is a specific type of ionizing radiation, characterized by slightly more complex, or ‘clustered’, DNA lesions compared to standard photon or X-ray radiation^34^. It has been hypothesized that unrepaired proton-induced DNA damage presents a greater obstacle to replication fork progression than X-rays but physical evidence for enhanced replication stress in proton-irradiated cancer cells has been lacking^35,36^.

Here, we set out to analyze the KRASmut replication stress phenotype in greater detail to uncover therapeutic liabilities. Using well characterized cell line models, we describe a baseline phenotype of replication stress and cytosolic DNA accumulation in untreated KRASmut cells that is unexpectedly resistant to exogenous stress. However, proton radiation specifically slows replication fork progression and increases fork stalling in KRASmut cells, suggesting a potential therapeutic opportunity to overcome the radioresistance associated with this tumor genotype.

## Results

### Increased replication stress and cytosolic dsDNA in untreated KRAS mutant cancer cells

**Figure 1:**
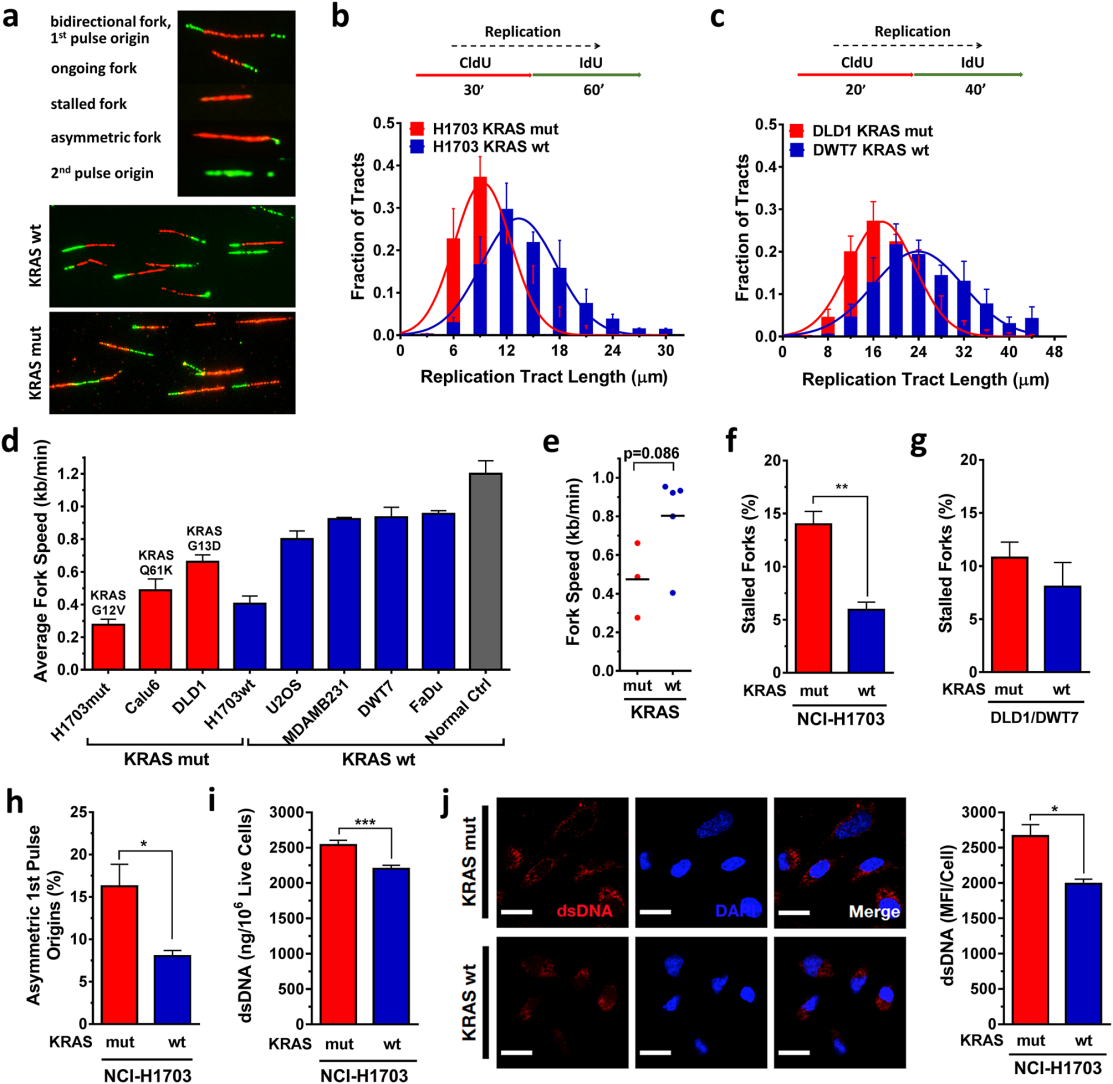
Baseline DNA replication stress and cytosolic DNA in KRAS mutant (mut) vs wild-type (wt) cancer cells. (**a**) Illustration of DNA fibers in exponentially growing cells that were pulse-labeled with thymidine analogues CldU and IdU and lysed after which DNA fibers were spread and immunodetected with specific antibodies against CldU and IdU. (**b, c**) Frequency distributions of DNA fiber length (i.e., CldU tract length) in isogenic cell pairs. (**d, e**) Velocity of fork progression in a panel of cancer cell lines (H1703, Calu-6 – lung cancer; DLD1/DWT7 – colon cancer; U2OS – osteosarcoma; MDA-MB231 – breast cancer; FaDu – head/neck cancer) and in a control (Ctrl) normal fibroblast line. (**f, g**) Percentage of fibers retaining only the first label (CldU) indicating fork stalling in isogenic cell pairs. (**h**) Percentage of bidirectional asymmetric forks that progressed through replication fork arrest. (**i**) Levels of double-stranded (ds) cytosolic DNA using PicoGreen dsDNA staining on cytosolic fractions from untreated cells. (**j**) Left, representative images of untreated cells stained with a dsDNA-specific antibody or DAPI. Right, semiquantification of fluorescent signal. All bars represent mean + /- SEM based on at least 3 independent repeats. Statistical comparisons by two-sided T-test indicating *p ≤ 0.05, **p ≤ 0.01, ***p ≤ 0.001.

To investigate the role of mutant KRAS in DNA replication stress, we visualized replication tracts and measured fork speed and structures using the DNA fiber method (Fig. 1a). Cells were pulse-labeled with thymidine analogues CldU and IdU and lysed, and DNA fibers were spread and immunodetected with specific antibodies against CldU and IdU. First, we assessed the fork speed of fiber tracts in two isogenic KRASmut and KRAS wild-type (wt) cell pairs, NCI-H1703 and DLD1/DWT7 cells^37^. In both cell lines, the distribution of replication tract lengths was consistent with a slowing in fork progression in the presence of KRASmut (Fig. 1b,c, Suppl. Fig. S1A,B). Similarly, in a non-isogenic comparison for which four cancer cell lines were added, the average fork speed trended slower in KRASmut cell lines (Fig. 1d,e).

Next, we counted fibers that retained only the first label (CldU) indicating fork stalling. We found KRASmut cells having more fork stalling compared to wt cells though this was less pronounced in the DLD1/DWT7 cell pair (Fig. 1f,g). In addition to fork slowing and stalling, bidirectional asymmetric forks are also associated with replication stress. These derive from asymmetrical fork progression through replication fork arrest. KRASmut cells had significantly more bidirectional asymmetric forks compared to wt cells (Fig. 1h). Lastly, to measure the new origin firing rate during the second labeling (second pulse origins), we counted the number of new origins fired during the IdU pulse (green fiber signal only) and divided it by the overall origin firing rate (calculated as the total number of origin firing events during the first (green–red-green fiber signal) and second labeling pulse (Fig. 1a)). KRASmut cells exhibited variable second pulse origin firing depending on cell line (Suppl. Fig. S1C, D).

DNA replication stress and damage can cause accumulation of genomic DNA fragments in the cytoplasm of cells^32,38^. We focused here on assessing dsDNA content in the cytoplasmic fraction of live untreated KRASmut and wt NCI-H1703 cells using PicoGreen dsDNA dye (Suppl. Fig. S2A-C). This revealed a statistically significantly 15% higher signal in KRASmut compared to wt cells (Fig. 1i). To confirm this observation, we stained untreated KRASmut and wt cells for cytoplasmic dsDNA using a specific dsDNA antibody and subsequently analyzed single-cell fluorescent intensity (Suppl. Fig. S2D). Again, KRASmut cells had on average significantly more dsDNA (34% higher signal) (Fig. 1j). While this data is correlative it is consistent with the notion that replication stress in KRASmut cells is linked to cytosolic dsDNA production. Taken together, our observations provide insight into the replication stress phenotype of KRASmut cells, which confirms and extends previous findings on the cellular effects of oncogenic KRAS^23,24^.

### Role of CHK1 kinase in regulating KRASmut-associated replication stress

**Figure 2:**
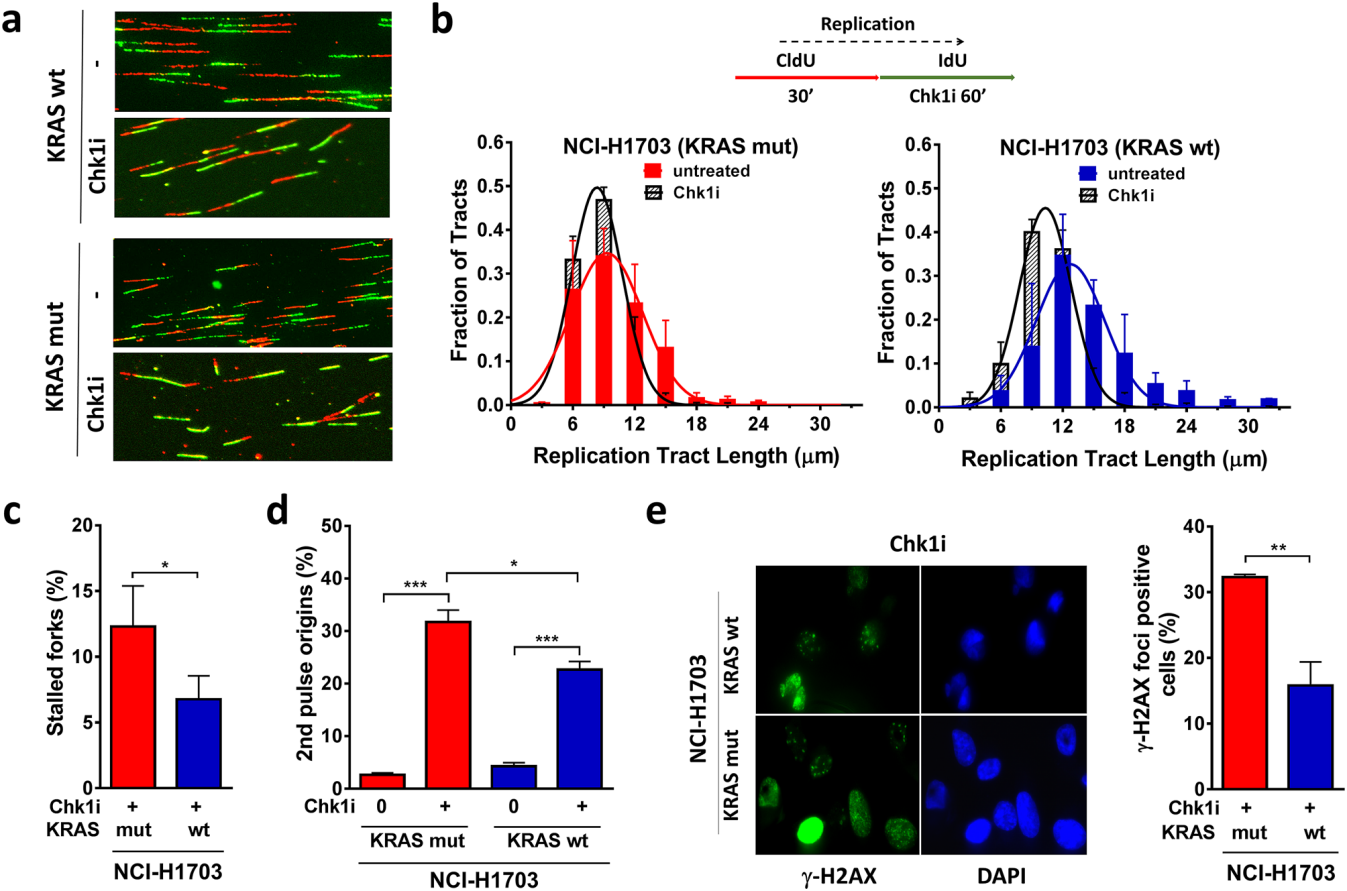
Impact of CHK1 kinase inhibition on replication stress in KRAS mutant (mut) vs wild-type (wt) cancer cells. (**a**) Representative immunofluorescence images depicting the effects of pharmacological CHK1 inhibition (Chk1i) with LY2603618 (1 µM) during the second label (IdU) in isogenic NCI-H1703 cancer cells. (**b**) Frequency distributions of DNA fiber length with and without LY2603618 treatment. (**c**) Percentage of fibers indicating stalled forks. (**d**) Percentage of the ratio of new origins fired during the second label (ldU) and overall origin firings during both pulses. (**e**) Left, representative immunofluorescence images showing nuclear γ-H2AX and DAPI staining in Chk1 inhibitor-treated cells. Right, percentage of cells with at least 5 γ-H2AX foci following 16 h of treatment with LY2603618. All bars represent mean + /- SEM based on 3 independent repeats. Statistical comparisons by two-sided T-test indicating *p ≤ 0.05, **p ≤ 0.01, ***p ≤ 0.001.

RAS signaling has been linked to activation of the ATR-CHK1 pathway^21,22,24,39^. We, therefore, asked whether inhibition of CHK1 with LY2603618 (Suppl. Fig. S3A) would worsen replication stress and potentially yield a therapeutic opportunity in our isogenic model. CHK1 inhibitor was added during the second labeling. Assessment of fork speed showed that inhibition of CHK1 did not cause a substantial decrease of fork progression (Fig. 2a,b) and only a minor stalling of forks in KRASmut cells compared to wt cells (Fig. 2c). Anticipating that CHK1 inhibition would disrupt CHK1-mediated suppression of origin firing, we analyzed the effect of CHK1 inhibitor treatment on replication initiation measuring second pulse origins. The percentage of second pulse origins increased in both KRASmut and wt cells, with a larger increase in the presence of KRASmut, which is consistent with a more pronounced baseline CHK1 activity in these cells (Fig. 2d). As a result of replication stress, DSBs may occur due to fork collapse. Cells were treated with CHK1 inhibitor for 16 h, which produced higher levels of DSBs as measured by γ-H2AX foci in KRASmut cells compared to wt cells (Fig. 2e). However, CHK1 inhibitor monotherapy did not cause selective cytotoxicity (Suppl. Fig. S3B), indicating that alternative therapeutic approaches are needed to target the replication stress phenotype of KRASmut cancer cells.

### Resistance of KRASmut cells to exogenous replication stressors

**Figure 3:**
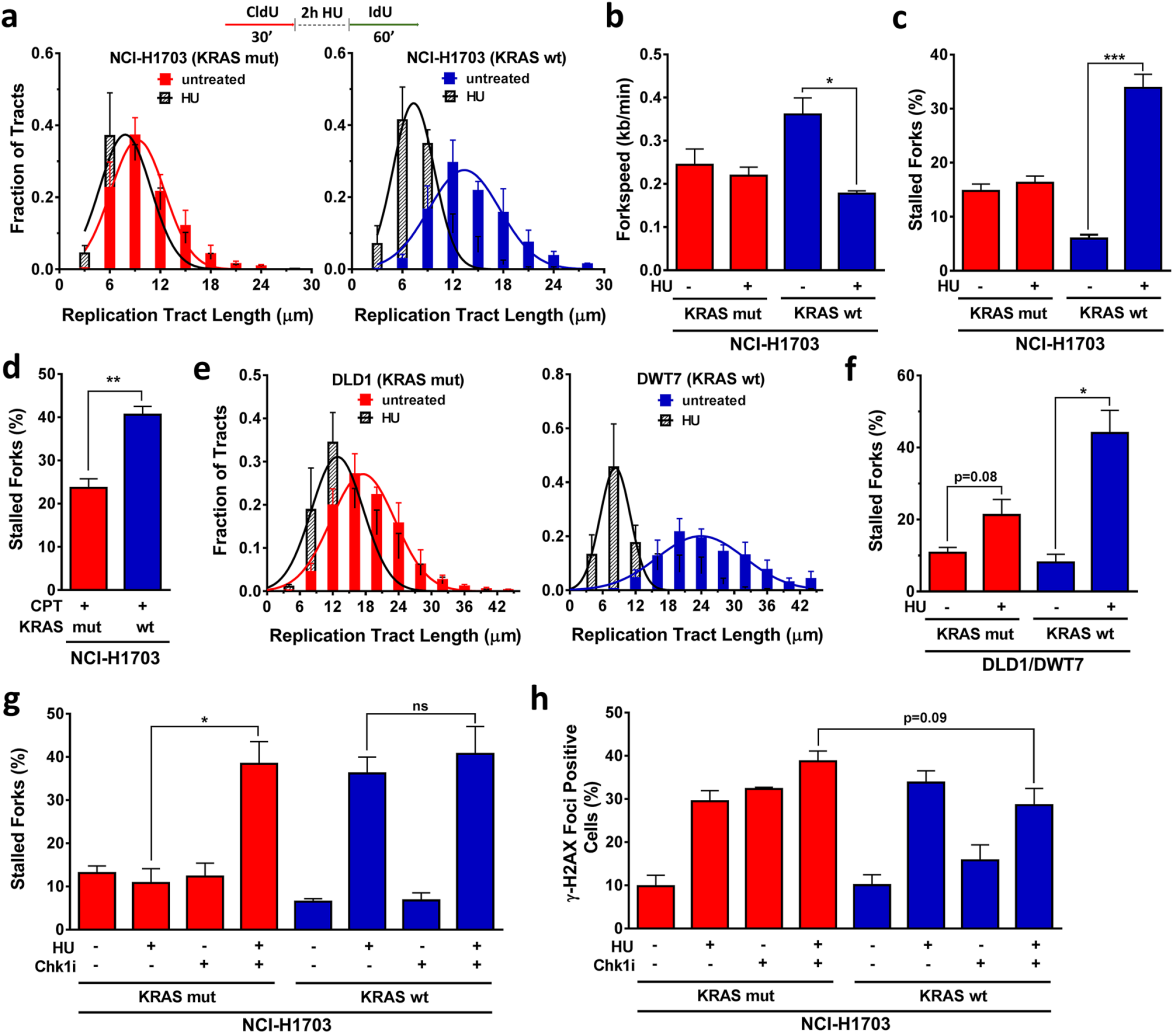
Resistance of KRAS mutant (mut) but not wild-type (wt) cancer cells to hydroxyurea (HU). (**a**) Distribution of fiber length in the isogenic NCI-H1703 pair with and without HU treatment (2 mM) administered for 2 h in between pulse labels. (**b**) Velocity of fork progression with and without HU treatment. (**c, d**) Percentage of fibers indicating fork stalling with/without 2 h treatment with HU or the topoisomerase I inhibitor camptothecin (CPT). (**e, f**) Distribution of fiber length and percentage of fork stalling in isogenic DLD1/DWT7 cells, analogous to panels A and B. (**g, h**) Combined effects of treatment with HU and CHK1 inhibitor (Chk1i) LY2603618 on fork stalling or γ-H2AX foci formation. All bars represent mean + /- SEM based on at least 3 independent repeats. Statistical comparisons by two-sided T-test indicating *p ≤ 0.05, **p ≤ 0.01, ***p ≤ 0.001; ns, not significant.

To determine whether endogenous replication stress in KRASmut cells could be therapeutically targeted, we treated cells with hydroxyurea (HU), which depletes cells of dNTPs initially resulting in stalled replication forks. Cells were treated with 2 mM HU for 2 h in between pulse labeling (Fig. 3a). In KRASmut cells, no further increase in replication stress above baseline was observed while in KRASwt cells HU led to significantly slower fork progression and increased fork stalling (Fig. 3a–c). To exclude the possibility that this finding was due to a KRAS effect on dNTP metabolism, we also treated cells with the topoisomerase I poison camptothecin (CPT). Similar to HU, CPT treatment led to greater exogenous replication stress in KRASwt cells than in KRASmut cells (Fig. 3d, Suppl. Fig. S3C-F). In addition, comparable observations were made in DLD1/DWT7 cells (Fig. 3e,f, Suppl. Fig. S3G).

Interestingly, CHK1 inhibition overcame the resistance of replication stress to HU in KRASmut cells (Fig. 3g, Suppl. Fig. S3G), which trended toward a correlation with higher levels of γ-H2AX foci after longer HU exposure (24 h) (Fig. 3h). However, when combining exogenous replication stress with CHK1 inhibition only mildly increased cytotoxicity was seen for KRASmut cells (Suppl. Fig. S3H). Taken together, despite their increased baseline replication stress KRASmut cells are largely resistant to exogenous replication stressors such as HU or CPT, which appears to be dependent at least in part on CHK1.

### Cytosolic dsDNA induction as a function of KRAS status

**Figure 4:**
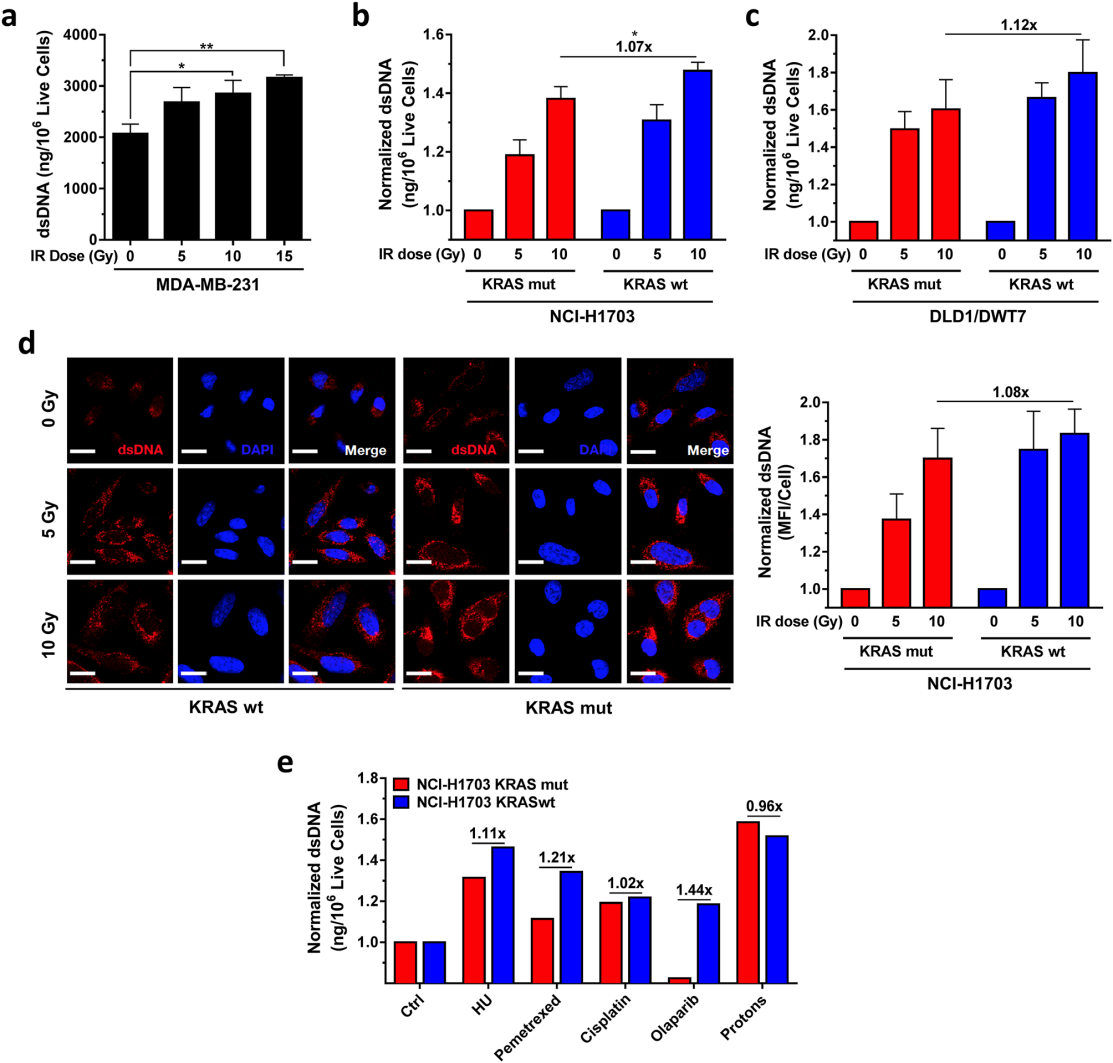
Response of cytosolic dsDNA to exogenous stressors in KRAS mutant (mut) and wild-type (wt) cancer cells. (**a**) Levels of dsDNA using PicoGreen dsDNA staining on cytosolic fractions from KRASmut MDA-MB-231 cancer cells 24 h after treatment with increasing single doses of ionizing radiation (IR), i.e., X-rays. (**b, c**) Normalized levels of cytosolic dsDNA in isogenic cell pairs. Fold-difference in dsDNA induction at the highest dose is shown. (**d**) Left, representative images of untreated cells stained with a dsDNA-specific antibody or DAPI. Right, semi-quantification of fluorescent signal. (**e**) Screen of isogenic NCI-H1703 cells with replication stress inducing anti-cancer treatments HU (2 mM), pemetrexed (0.2 µM), cisplatin (20 µM), olaparib (5 µM), and proton radiation (10 Gy) showing normalized levels of dsDNA based on 1–6 repeats. Bars represent mean + /- SEM based on 3 independent repeats except where indicated. Statistical comparisons by two-sided T-test indicating *p ≤ 0.05, **p ≤ 0.01, ***p ≤ 0.001 (absence of these indicators signifies *p* > 0.05).

Next, we asked whether the observed resistance of KRASmut cells to exogenous replication stressors would also result in a blunted cytosolic dsDNA production. We initially utilized radiation treatment with X-rays, which not only is linked to replication stress but also acts a potent inducer of cytosolic DNA^27,33^. To confirm our ability to detect radiation-induced cytosolic DNA, we first reproduced the radiation dose response that was seen in MDA-MB-231 cells in a recent study by Sylvia Formenti and colleagues, as shown in Fig. 4a^33^. Similarly, we found that 10 Gy irradiation caused on average a 1.4-fold higher dsDNA signal compared to baseline. A comparable 5 and 10 Gy dose response was seen in NCI-H1703 cells (Fig. 4b), yet the induction of dsDNA was less pronounced in the KRASmut clone compared to wt cells, i.e., statistically significant 1.07-fold lower induction after 10 Gy (Fig. 4b). At the same time, replication fork progression in KRASmut cells was not slowed by radiation in contrast to KRASwt cells (Suppl. Fig. S4A). A similar observation was made in KRASmut DLD1 cells in which cytosolic dsDNA induction was 1.12-fold lower than in wt cells following 10 Gy irradiation, though this difference did not reach statistical significance (Fig. 4c). A second assay in NCI-H1703 cells also found a numerically lower dsDNA induction of 1.08-fold (Fig. 4d).

We next screened additional replication-targeted agents to further confirm the reduced ability of KRASmut cells to induce cytosolic dsDNA in response to genotoxic treatments, which appeared to correlate with the observed exogenous replication stress resistance (Fig. 3). Treatment with different anti-cancer agents, including HU, again revealed numerically lower dsDNA induction in KRASmut cells compared to wt cells (range, 1.02–1.44-fold) (Fig. 4e). Interestingly, proton radiation appeared to be an exception to this (Fig. 4e) although we note that the differences in this screen were not associated with statistical significance.

### Proton radiation targets the replication stress phenotype of KRASmut cells

**Figure 5:**
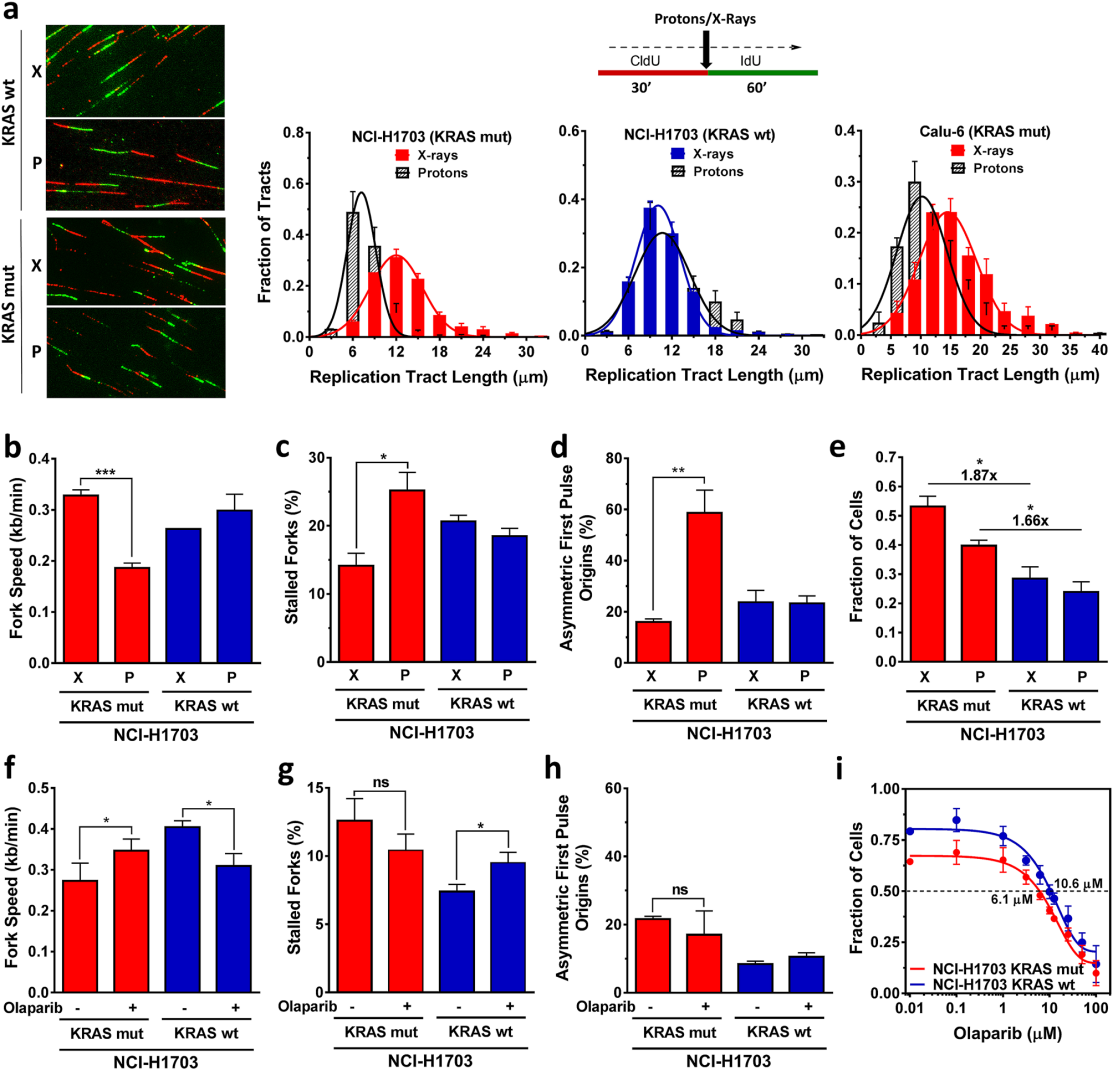
Cellular response of KRAS mutant (mut) and wild-type (wt) cancer cells to treatment with radiation or PARP inhibition (PARPi). (**a**) Left, representative images illustrating single DNA fibers following irradiation with 2 Gy X-rays (X) or protons (P) delivered in between pulse labels. Right, distribution of fiber length in irradiated KRASmut and wt cells. (**b–d**) Velocity of fork progression, percentage of stalled forks, and percentage of bidirectional asymmetric first pulse origins in isogenic cells irradiated with 2 Gy. (**e**) Fraction of cells surviving 5 days following irradiation with 6 Gy using a validated 3D viability assay^14^. (**f–h**) Velocity of fork progression, percentage of stalled forks, and percentage of bidirectional asymmetric first pulse origins in isogenic cells treated with/without PARPi olaparib (5 µM). (**i**) Fraction of cells surviving after 5 days of treatment with varying concentrations of olaparib. The drug concentration to achieve 50% cell survival (IC50) is indicated. All bars represent mean + /- SEM based on 2–3 independent repeats. Statistical comparisons by two-sided T-test indicating *p ≤ 0.05, **p ≤ 0.01, ***p ≤ 0.001; ns, not significant.

Data from us and others have suggested that proton-induced complex DNA damages are particularly toxic to cancer cells with defects in homologous recombination and associated repair pathways^35,36,40–42^. However, the effects of proton irradiation on cells exhibiting replication stress are unknown. Given the unexpected finding in Fig. 4e with regard to the ability of proton radiation to induce cytosolic dsDNA in KRASmut cells, we returned to study the replication response of KRASmut and wt cells to proton treatment. Cells were treated with 2 Gy X-rays or protons between CldU and IdU labeling (Fig. 5a). Proton treatment conditions did not affect the replication stress phenotype of KRAS mut cells (Suppl. Figure 4B,C). We also previously confirmed that cell growth was not affected by the experimental set up^35^. The distribution of fiber tract lengths in KRASmut NCI-H1703 cells revealed a markedly more pronounced shortening in length after proton than after X-irradiation (Fig. 5a). In contrast, in KRASwt cells the distribution curves almost completely overlapped. A comparable differential effect of proton vs X-ray treatment was seen in KRASmut Calu-6 cells (Fig. 5a, Suppl. Fig. S4D). Consistent with these findings there was reduced fork speed, increased fork stalling, and asymmetric fork progression after proton irradiation in both KRASmut NCI-H1703 and Calu-6 cell lines (Fig. 5b–d, Suppl. Fig. S4E, F). Furthermore, there also appeared to be a differential dependence on CHK1 after treatment with X-rays versus protons (Suppl. Fig. S5A-C) though the exact functions of CHK1 in the response to damage from these two radiation modalities remain to be elucidated.

Next, we asked whether increased replication stress following proton irradiation might correlate with enhanced radiosensitivity. KRASmut status is associated with radioresistance in vitro and in vivo as previously described by us and others^13–16,43,44^. Accordingly, both X-ray and proton irradiation was more toxic in the presence of KRASwt (Fig. 5e). Interestingly, the radioresistance of KRASmut cells appeared somewhat less pronounced after protons than after X-rays, i.e., 1.66-fold vs 1.87-fold more resistant than wt cells. However, the data also suggests that replication stress associated with proton irradiation was not sufficient to fully overcome the radioresistance of KRASmut cells. Taken together, our data indicates a different biology following proton vs. X-ray treatment that is revealed in the setting of pre-existing replication stress in KRASmut cells.

Proton irradiation and PARP1 inhibition have preferential cytotoxic effects in cells with defects in certain DNA repair pathways such as homologous recombination^35,36^. However, as DNA damages caused by these two treatments are different we sought to compare the replication response to protons with that to the PARP inhibitor olaparib. Interestingly, in KRASmut cells olaparib increased fork speed and did not induce fork stalling or bidirectional asymmetric fork progression, as reported in other cell models^45^, while the opposite was seen in KRASwt cells (Fig. 5f–h). Olaparib was mildly more toxic to KRASmut cells compared to wt cells (Fig. 5i) but overall these cells remained essentially resistant to PARP inhibition when compared to homologous recombination-defective cell lines (not shown). Lastly, recent data suggested that combined CHK1 and PARP inhibitor treatment is preferentially toxic to KRASmut HTC116 cells^23^; however, we did not observe any differential cell kill when these inhibitors were combined, possibly highlighting inter-tumoral differences across KRASmut cancers (Suppl. Fig. S5D).

## Discussion

We detail here the DNA replication stress phenotype that associates with oncogenic KRAS in isogenic and non-isogenic cancer cell line models. The majority of the data was obtained in NCI-H1703 NSCLC cells expressing KRASmut, which have been previously confirmed to be a clinically relevant model for KRASmut associated radioresistance in vitro and *in vivo*^14,43^. Using the single-molecule DNA fiber assay we found a higher level of replication fork stalling and slowed fork progression in untreated KRASmut cells compared to KRASwt cells. Our data are consistent with and extend prior reports that oncogenic RAS signaling and other factors result in replication stress and increased CHK1 activity that may be protective (Figs. 1, 2, 3)^21,23,24,39,46^. However, KRASmut cells did not appear to be significantly more sensitive to pharmacologic CHK1 inhibition than wt cells, even when combined with replication-targeted treatments such as a topoisomerase I poison (CPT) or a PARP1/2 inhibitor (olaparib) (Fig. 5i, Suppl. Fig. S3H, S5D). Other recent studies have reported interesting KRASmut- and replication-specific targets that may have translational potential including MCM7-dependent replication licensing and the AATF transcription factor^47,48^.

While the reasons for the observed resistance of KRASmut cells to exogenous replication stressors such as HU or CPT remain unresolved, we unexpectedly found that proton radiation was able to further enhance the baseline replication stress in KRASmut cells compared to KRASwt cells (Fig. 5a–d). Differential cellular responses to proton radiation as opposed to photons or X-rays are still poorly understood^34^. Protons produce slightly more complex DNA damages than X-rays which can be potentially exploited for therapeutic purposes. The available data, based on indirect repair foci and genetic analyses, have suggested that complex DNA damage induced by proton radiation challenges the progression of replication forks, thereby leading to an increased dependency on homologous recombination and Fanconi Anemia (FA) genes for DNA repair and replication fork restart^35,36,40–42^. However, direct data for interference with fork progression has been lacking. We provide here physical evidence that protons specifically slow fork progression and increase fork stalling to a greater degree than X-rays in repair-proficient KRASmut NCI-H1703 as well as FA-defective Calu-6 cells. In contrast, there was no differential effect of protons vs X-rays in KRASwt cells. The consequences of proton irradiation in KRASmut cells clearly contrast those of other drugs that affect replication such as CPT, HU, or olaparib, highlighting the unique nature of the underlying DNA damage.

The radioresistance of NCI-H1703 cells expressing KRASmut and its underlying mechanisms have been reported^14,37,43^. However, how the replication stress phenotype of these cells may relate to their radioresistance is unknown. Interestingly, in a glioblastoma model, replication stress induced radioresistance, presumably through constitutive activation of the DNA damage response^49^. In this context, it is noteworthy that CHK1 inhibition preferentially radiosensitizes KRASmut cells though how much of that effect is caused by modification of replication stress remains to be elucidated^50,51^. Further, replication stress and radioresistance in relation to cancer stem cell-like phenotypes require additional study^14,52^.

The observed enhancement of replication stress by proton treatment appears to be correlated with increased cytotoxicity in the NCI-H1703 model where proton treatment was associated with less pronounced radioresistance than standard X-rays in KRASmut cells compared to wt cells (Fig. 5e). However, protons could only partially overcome KRASmut radioresistance, suggesting an opportunity to develop KRASmut-specific combinations of protons with replication-targeted agents. While we used a validated cell viability assay in Fig. 5e, we caution that additional study will be needed to determine the relative biological effectiveness of protons in KRASmut cells using standard clonogenic survival assays on our NCI-H1703 cells and additional models.

DNA replication stress has been linked to the release of genomic DNA into the cytoplasm^32,38^. For example, in cells with impaired RAD51 recombinase function, excessive nucleolytic degradation of newly replicated DNA is a major source of cytosolic DNA production and innate immunity in response to replication stress induced by radiation^32^. In contrast, how replication stress in KRASmut cells, which have intact or upregulated RAD51^23^, results in cytosolic DNA generation has not been described. Our data show that while cytosolic DNA is readily induced in KRASmut cells by different exogenous stressors including ionizing radiation (Fig. 4), the underlying baseline replication stress does not translate into higher levels of cytosolic DNA compared to KRASwt cells. If anything, cytosolic DNA levels trend lower than in KRASwt cells, with the exception of proton radiation. These findings mirror the resistance of KRASmut cells to exogenous inducers of replication stress. We acknowledge that the link between replication stress and cytosolic DNA production in our study remains correlative at this point. However, our data provide a foundation for future studies to elucidate common regulators of replication stress and cytosolic DNA in KRASmut tumors that could be therapeutically exploited.

Taken together, our data highlight the challenges that the treatment-resistant KRASmut cellular phenotype poses. We provide physical evidence for a differential effect of proton radiation targeting DNA replication stress in cancer cells driven by the KRAS oncogene, which may yield a novel therapeutic opportunity. Replication stress in these tumors remains an attractive target for cancer- and genotype-selective therapies. Lastly, as there clearly exists treatment-relevant inter-tumoral heterogeneity among KRASmut cancers^18^, we wish to stress that the findings of this study should not be generalized beyond the preclinical models employed here.

## Methods

### Cell lines

Cell lines and cell culture conditions have been described^35,37,50^. The identity of each cell line was authenticated as described previously^53^, and additional cell line authentication was performed by Bio-Synthesis, Inc. No cell line was ever treated for mycoplasma and all lines tested mycoplasma free with the MycoAlert Kit (LONZA, Cat #LT07-218) before experiments.

### Treatments

Chk1 inhibitor (LY2603618, Cat #S2626) was purchased from SelleckChem. Hydroxyurea (Cat #H8627), camptothecin (Cat #C9911), and cisplatin (Cat #P4394) were purchased from Sigma-Aldrich. Pemetrexed (Cat #P-7177) and olaparib (AZD-2281; Cat #O-9201) were purchased from LC Laboratories. Drugs were aliquoted and stored according to the manufacturer’s instructions. Treatment with orthovoltage X-rays was performed using a PXi Precision X-Ray X-RAD 225 Lite X-ray generator operated at 225 kV (0.5 mm Cu HVL) and 13.30 mA, at a dose rate of 2.08 Gy/minute. Proton irradiation was performed using the clinical proton beam (235 meV) at the Francis H. Burr Proton Therapy Center at Massachusetts General Hospital, as described previously^35^. Briefly, irradiation was performed with a passively scattered beam with a range of 13.4 cm (90%) and modulation width of 7 cm (90%-98%). We used the largest snout with an effective field size of 22 cm in diameter within 2% of dose uncertainty. A Lucite phantom was used to position cell culture vessels at the center of the spread-out Bragg peak, that is, at 10.3 cm water equivalent depth from the front surface of the phantom. The LET at this position was 2.5 keV/mm, and the dose rate was 1.7 Gy/min. Physical doses without RBE correction were used. For the DNA fiber assay, cells were placed on ice after the first label and subjected to proton irradiation (in parallel to X-irradiation under the same conditions), and following return to the laboratory the second labeling was carried out.

### DNA fiber assay

The fiber assay was carried out essentially as described previously^26,46,54^. Exponentially growing cells were pulse-labeled with 25 µmol/L 5-chloro-2-deoxyuridine (CldU, Cat #C6891, Sigma-Aldrich) followed by 250 µmol/L 5-iodo-2-deoxyuridine (IdU, Cat #I7125, Sigma-Aldrich) as indicated. Labeling was stopped with ice-cold phosphate-buffered saline (PBS). Cells were collected and suspended at a concentration of 500,000/ml. A 2.5 µl suspension was applied to a glass slide and mixed with 7.5 µl spreading buffer (200 mM Tris-HCl pH 7.4, 50 mM EDTA, 0.5% SDS). Slides were angled at 15° to spread the DNA molecules. Dry-spread DNA was fixed in methanol-acetic acid (3:1) for 10 min and slides were stored at 4 °C overnight as soon as dried. Slides were rehydrated with double distilled water and incubated for 75 min at room temperature in 2.5 M HCl. Slides were then washed with PBS and incubated in blocking solution (3% BSA in PBS, 0.1% Tween) for 1 h. BrdU rat and mouse monoclonal antibodies (Cat #ab6326, Abcam and Cat #347580, BD Biosciences) were used for CldU and IdU detection, respectively. Primary antibodies at a concentration of 1:500 in blocking solution were applied to the slides at 37 °C for 2 h. DNA fiber spreads were fixed for 10 min with 4% PFA and washed with PBS. Secondary antibodies goat anti-rat IgG Alexa Fluor 555 (Cat #A-21434, Invitrogen) and goat anti-mouse IgG Alexa Fluor 488 (Cat #A-11029, Invitrogen) were applied 1:500 in blocking solution to the slides for 1.5 h at room temperature. Slides were mounted in Vectashield Vibrance mounting medium (Vector Laboratories, Cat #H1700). CldU and IdU lengths were measured with ImageJ. For counting fiber structures, the Cell Counter Plug-in of ImageJ was used. Immunofluorescent images (60X, Nikon 90i) were taken from randomly selected fields with untangled fibers and analyzed. For structure analyses, the frequencies of the different classes of fiber tracks were classified: red-green (ongoing replication tracts), red (stalled forks/termination), green-red-red-green (1st order origin) and green (2nd order origin). For fork speed analyses, replication fork speeds of CldU and IdU were measured and micrometer values were converted into kilobases. A conversion factor of 1 µm = 2.59 kb was used^55^. A minimum of 100 individual fibers was analyzed for each experiment, and the means of at least 3 independent repeat experiments were calculated.

### Cytosolic dsDNA measurements

Quantification of cytosolic dsDNA in cytosolic extract was, with minor modifications, performed as described^32,33^. Cells were grown until 80% confluent and treated with drug or radiation. Cells were treated for 24 h with the indicated drug. Cells treated with irradiation were incubated for 24 h after exposure. The concentration of live cells in suspension was determined using a Cellometer K2 (Nexcelom) and the AO/PI Viability assay (Nexcelom Bioscience, Cat #CS2-0106). Cytoplasmic extract from 10^6^ live cells was generated using NE-PER Nuclear and Cytoplasmic Extraction Kit (Thermo Scientific, Cat #78833). The supernatant containing the cytosolic extract was either immediately used or stored at − 80 °C until use. dsDNA in cytoplasmic fractions was quantified using the Quant-iT PicoGreen dsDNA Assay Kit (Invitrogen, Cat #P7589). Black 96-well plates (Greiner Bio-One, Cat #655076) were loaded with 98 µl of assay buffer and 2 µl of cytosolic extract. Subsequently, 100 µl of aqueous PicoGreen working solution was added, and plates were mixed and incubated according to manufacturer’s instructions. The samples were excited at 480 nm and the fluorescence emission intensity was measured at 520 nm using a SpectraMax i3x Microplate Reader (Molecular Devices). Standard curves to calculate final dsDNA concentration were generated for each experiment using the given lambda dsDNA standard.

### Immunofluorescence microscopy

Cells were grown on cover slides and treated with varying doses of ionizing radiation. After 24 h, cells were rinsed in PBS, fixed in 4% PFA for 20 min at room temperature followed by three washes with PBS. Cells were permeabilized with 0.2% Triton-X in PBS for 10 min at room temperature followed by three washes with PBS + 0.1% Tween20 (PBST). For selective DNA denaturization, cells were treated with 50% formamide for 10 min at room temperature followed by treatment with 50% formamide for 15 min at 75 °C and three washes with TBS. Next, cells were treated with RNAseA (1 mg/ml, Invitrogen PureLink RNaseA, Cat #12091-02) for 1 h at 37˚C and blocked for 1 h at room temperature with 1% BSA and 2% goat serum in PBS. Samples were then incubated with primary dsDNA antibody (10 µg/ml or 1:100; MAB1293, Chemicon) at 4 °C overnight, washed three times with PBST, incubated with Cy3 conjugated secondary antibody (5 µg/ml or 1:200; AP124C, EDM Millipore), again washed three times with PBST, stained with 0.4 µg/ml DAPI in PBS, and subsequently mounted in Vectashield Vibrance mounting medium (Vector Laboratories, Cat #H1700). Images were acquired using an Olympus FV1000 confocal laser scanning microscopy system (Olympus, Cat #F10PRDMYR-1) and fluorescent intensity per cell was quantified for more than 100 cells per condition and experiment using Image J.

For γ-H2AX foci measurements, cells were fixed with 4% PFA for 15 min and then permeabilized with PBS containing 0.5% Triton-X for 20 min at 4 °C. Cells were blocked for 1 h at room temperature with 5% goat serum in PBS. Primary antibody against γ-H2AX (1:1000; JBW301, Millipore, Cat #05-636) was added for 2 h at room temperature in PBS containing 3% goat serum and 0.1% Triton-X. Subsequently probes were incubated with fluorescent secondary antibody Alexa Fluor 488 (1:500; Invitrogen, Cat #A-11029) in PBS containing 3% goat serum and 0.1% Triton-X for 1 h at room temperature. Nuclei were counterstained with DAPI and subnuclear protein foci were scored by fluorescence microscopy using an Olympus BX51 microscope.

### Cell survival assays

Cell survival assays were carried out as previously described^35,56^. Briefly, exponentially growing cells were seeded into 96-well plates with optimized cell density for 2D culture or tumor sphere formation, and treated with different drug concentrations for 5 days or incubated for 5 days following 8 Gy irradiation, respectively. Fractions of viable cells were determined using the CellTiter-Glo Luminescent Cell Viability Assay (Promega). Signals were read using a SpectraMax M5 Microplate Reader (Molecular Devices).

## Supplementary Information

Supplementary Information
