# Supplementary material for: Targeting the DNA replication stress phenotype of KRAS mutant cancer cells
Source: Sci Rep. 2021 Feb 11;11:3656. doi: 10.1038/s41598-021-83142-y (PMC7878884; doi:10.1038/s41598-021-83142-y)
Supplement: Supplementary file 1 — Supplementary Information [file 41598_2021_83142_MOESM1_ESM.pdf]

Supplementary Data

Targeting the DNA Replication Stress Phenotype of KRAS Mutant Cancer Cells

Tara Al Zubaidi[1,2*], O. H. Fiete Gehrisch[1,2*], Marie-Michelle Genois[3], Qi Liu[1,4], Shan Lu[1,5], Jong Kung[1], Yunhe Xie[1], Jan Schuemann[1], Hsiao-Ming Lu[1], Aaron N. Hata[3], Lee Zou[3], Kerstin Borgmann[2**], and Henning Willers[1**]

[1]Department of Radiation Oncology, Massachusetts General Hospital, Harvard Medical School, Boston, Massachusetts
[2]Laboratory of Radiobiology and Experimental Radiooncology, Clinic of Radiotherapy and Radiooncology, University Medical Center Hamburg-Eppendorf, Hamburg, Germany
[3]Center for Cancer Research, Massachusetts General Hospital, Harvard Medical School, Charlestown, Massachusetts
[4]Shenzhen Bay Laboratory, Shenzhen, China
[5]Harbin Medical University Cancer Hospital, Harbin, China
*co-first authors, **co-senior authors

Supplementary Figures: 5
Tables: 0

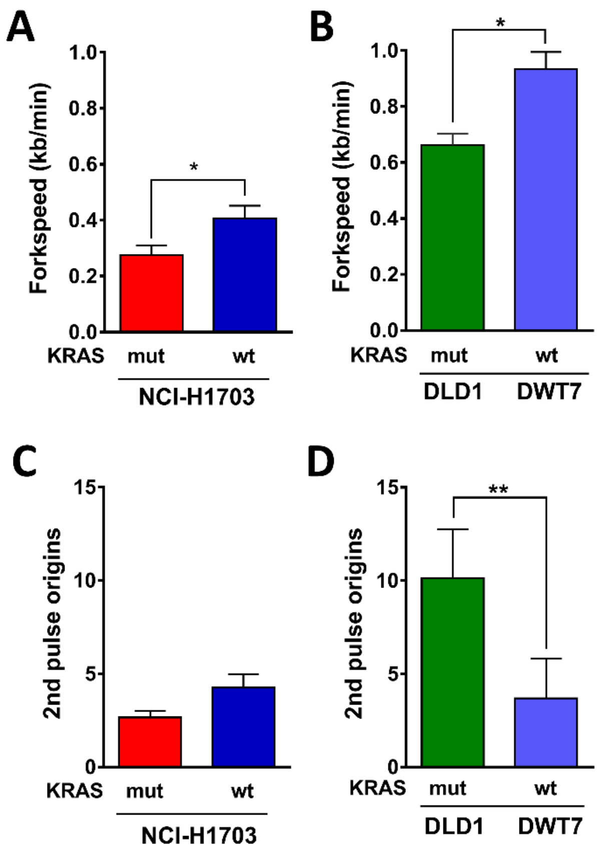

Supplementary Figure S1. Replication stress in KRAS mutant (mut) and wild-type (wt) cancer cells. (**A, B**) Velocity of fork progression in isogenic cell pairs. (**C, D**) Relative number of new origins fired during the IdU pulse and divided by the overall number of origin firings. All bars represent mean +/- SEM based on 3 independent repeats. Statistical comparisons by two-sided T-test indicating * p≤0.05, ** p≤0.01, *** p≤0.001.

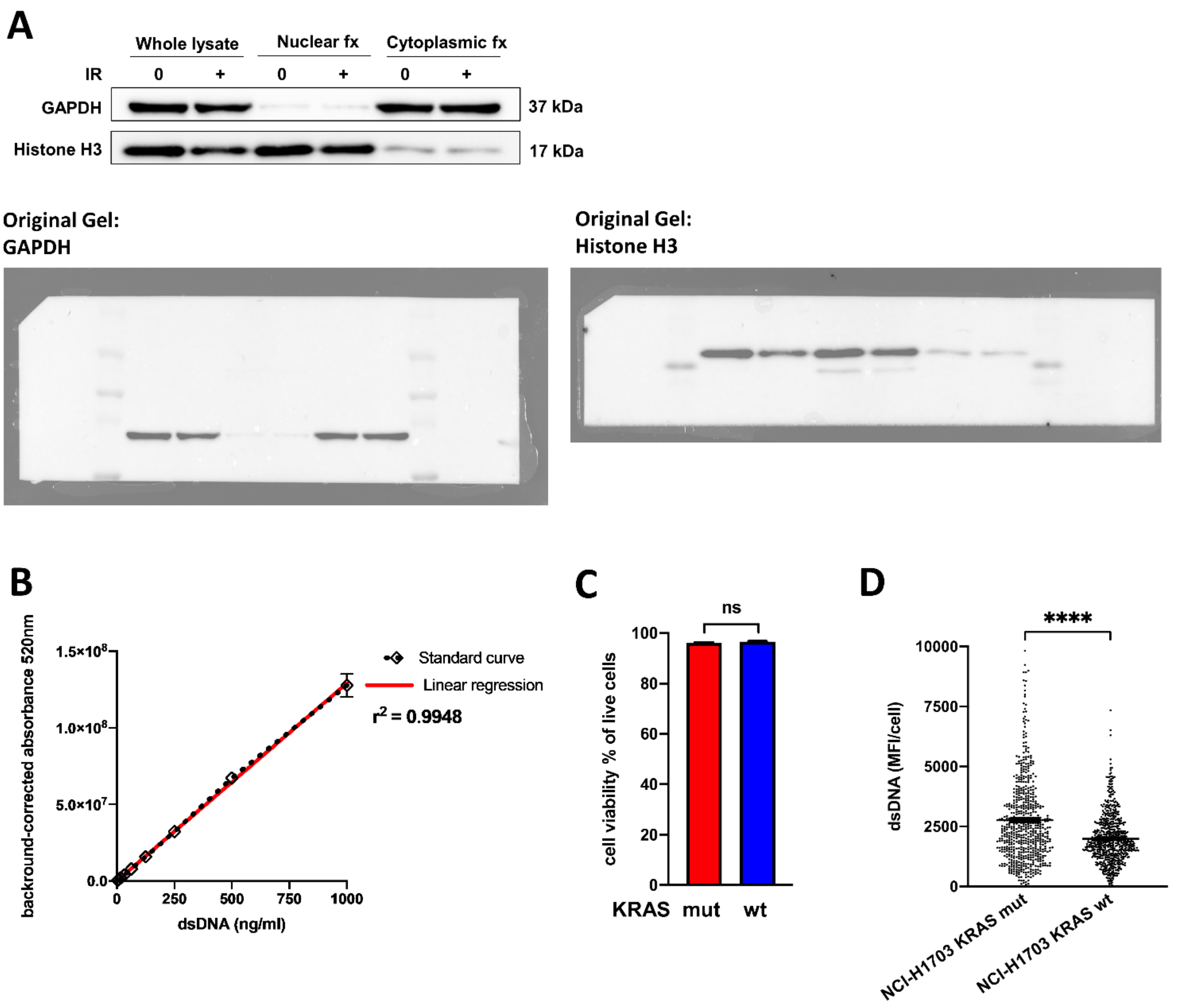

Supplementary Figure S2. Determination of cytosolic double-stranded (ds) DNA. (**A**) Upper Image, Confirmation of purity of cytosolic fraction. Whole cell lysates, nuclear fraction (fx), and cytoplasmic fx from NCI-H1703 cells were prepared with extraction buffer (Thermo Scientific, Cat #FNN0011) and NE-PER Nuclear and Cytoplasmic Extraction Kit (Thermo Scientific, Cat # 78833), both containing Halt Protease Inhibitor Cocktail (Thermo Scientific, Cat #87786). Protein concentrations were determined using Bio-Rad Protein Assay Dye (BioRad, Cat #500-0006). Western blotting was carried out using standard procedures. Briefly, a total of 30-50 µg protein of each sample was loaded in a 4-12% Bis-Tris Gel (Invitrogen, Cat #NP0321) and run for 30 min at 100V and for 60-120min at 150V on ice. Transfer of proteins on an activated PVDF membrane (Thermo Scientific, Cat # LC2002) was performed for 70 min at 100V at 4°C. Membranes were probed with primary antibodies for GAPDH (1:5000; D16H11, Cell Signaling Technology, Cat #5174) and Histone H3 (1:2000; D1H2, Cell Signaling Technology, Cat #4499) in 0.1% 1XTBS with 0,1% Tween20 (TBST) and 5% nonfat dry milk at 4°C overnight and washed three times with TBST. Blots were then incubated with Horseradish peroxidase–conjugated secondary antibody (1:5000; Santa Cruz Biotechnology, Cat #sc-2357), TBST and 5% nonfat dry milk for 1h at room temperature and washed three times with TBST. Protein bands were visualized using chemiluminescence (Bio-Rad Laboratories, Cat #170-5060) and images were acquired using the ChemiDoc MP Imaging System (Bio-Rad Laboratories). The same blot was used for visualizing both proteins. No image processing was performed. Lower Images, original gel images (left, 17h42min48sec; right,

18h12min13sec). (**B**) To ensure PicoGreen assay robustness, the provided standard fluorescent readouts were plotted, and the calculated linear regression shows reliable linearity of readout and standard concentration. (**C**) Nuclear contamination of cytoplasmic fractions due to increased cell death and subsequent apoptosis was ruled out by performing the AO/PI Viability assay. (**D**) NCI-H1703 KRASmut and wt cells were stained with a dsDNA specific antibody and the cytoplasmic staining signal per cell was quantified.

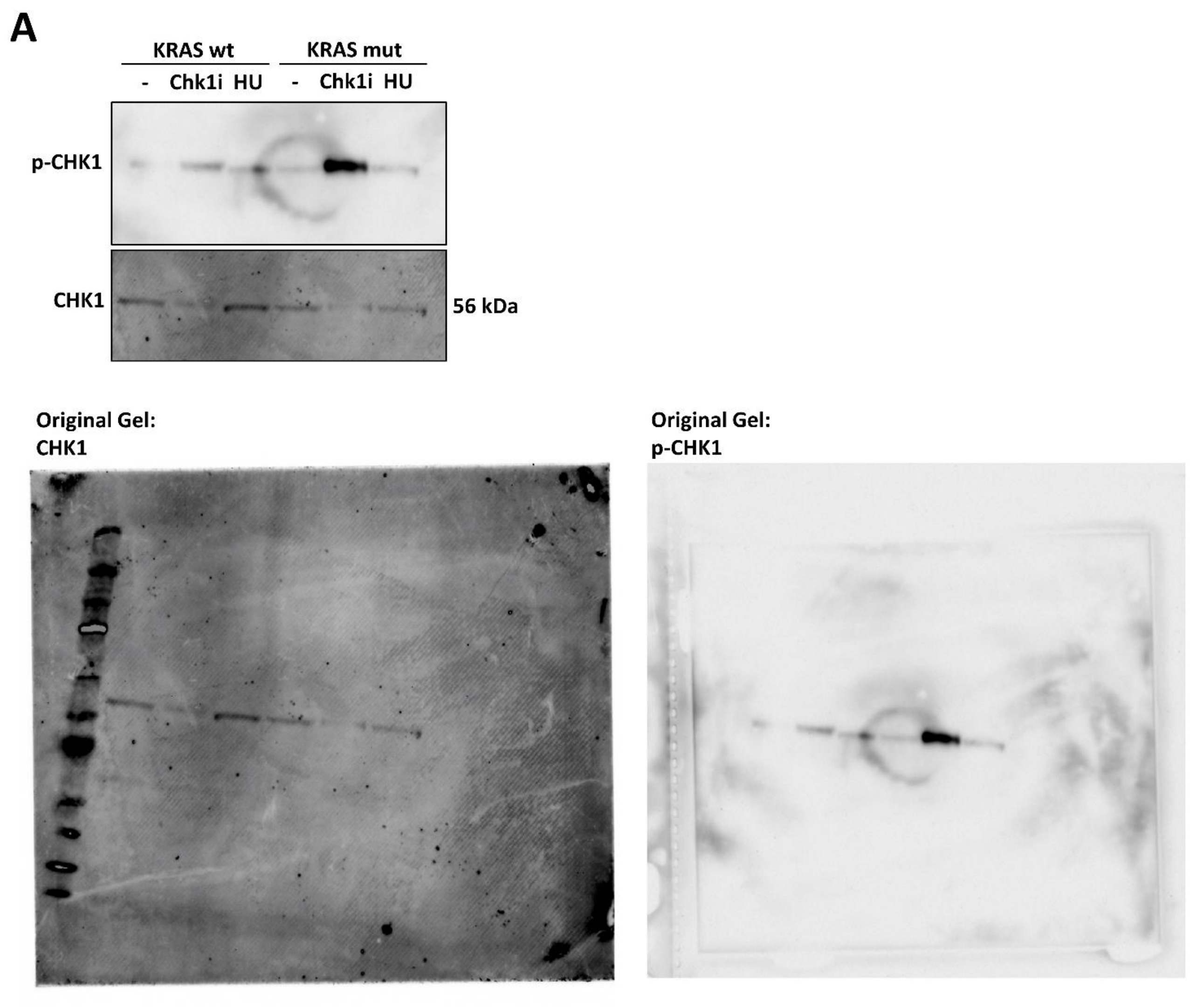

Supplementary Figure S3. Exogenous replication stress and CHK1 function in KRAS mutant (mut) and wild-type (wt) cancer cells. (**A**) Upper Image, Western blot on whole cell lysates from NCI-H1703 cells depicting expected increase in phospho-S345 on CHK1 (Cell Signaling, #2341) following treatment with LY2603618 (1 μM) (as described in Wang et al., Apoptosis 2014 Sep;19(9):1389-98) with HU treatment as a control for damage-induced S345 phosphorylation. Chemiluminescence images were acquired as described for Fig. S2A. No image processing was performed. Lower Images, original gel images (left, 15h10min39sec; right, 11h00min31sec).

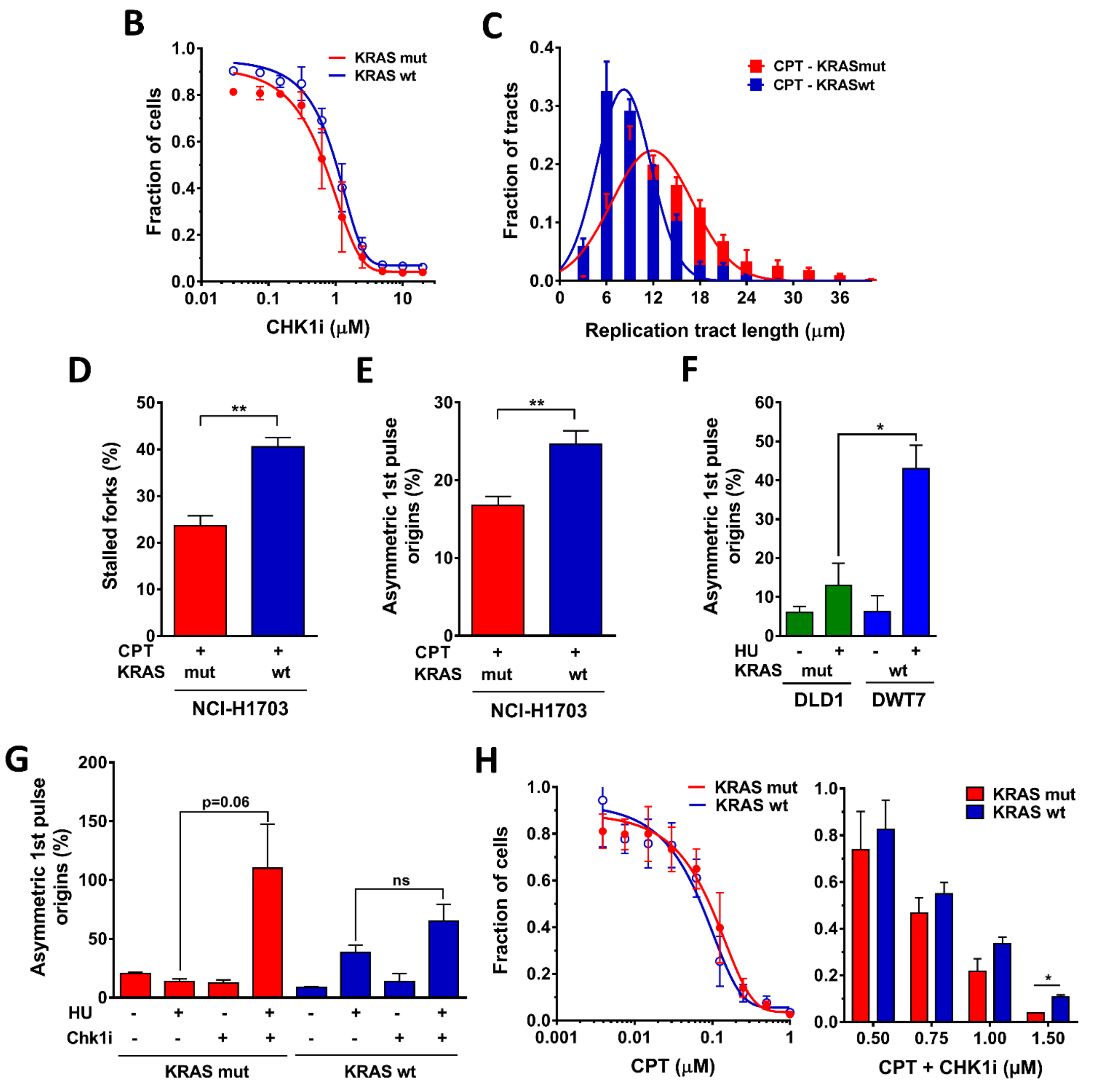

Supplementary Figure S3 continued. Exogenous replication stress and CHK1 function in KRAS mutant (mut) and wild-type (wt) cancer cells. (**B**) Fraction of NCI-H1703 cells after 5 days of CHK1 inhibitor LY2603618 treatment. (**C**) Distribution of fiber tract length following treatment with camptothecin (CPT) in between pulse labels. (**D**) Percentage of stalled replication forks. (**E-G**) Percentage of bidirectional asymmetric forks that progressed through replication fork arrest. (**H**) Left, fraction of NCI-H1703 cells after 5 days of CPT treatment. Right, combined CHK1 inhibitor LY2603618 treatment plus CPT at IC70 dose. All data points represent mean +/- SEM based on 2-3 independent repeats. Statistical comparisons by two-sided T-test indicating * $p \leq 0.05$, ** $p \leq 0.01$, *** $p \leq 0.001$; ns, not significant.

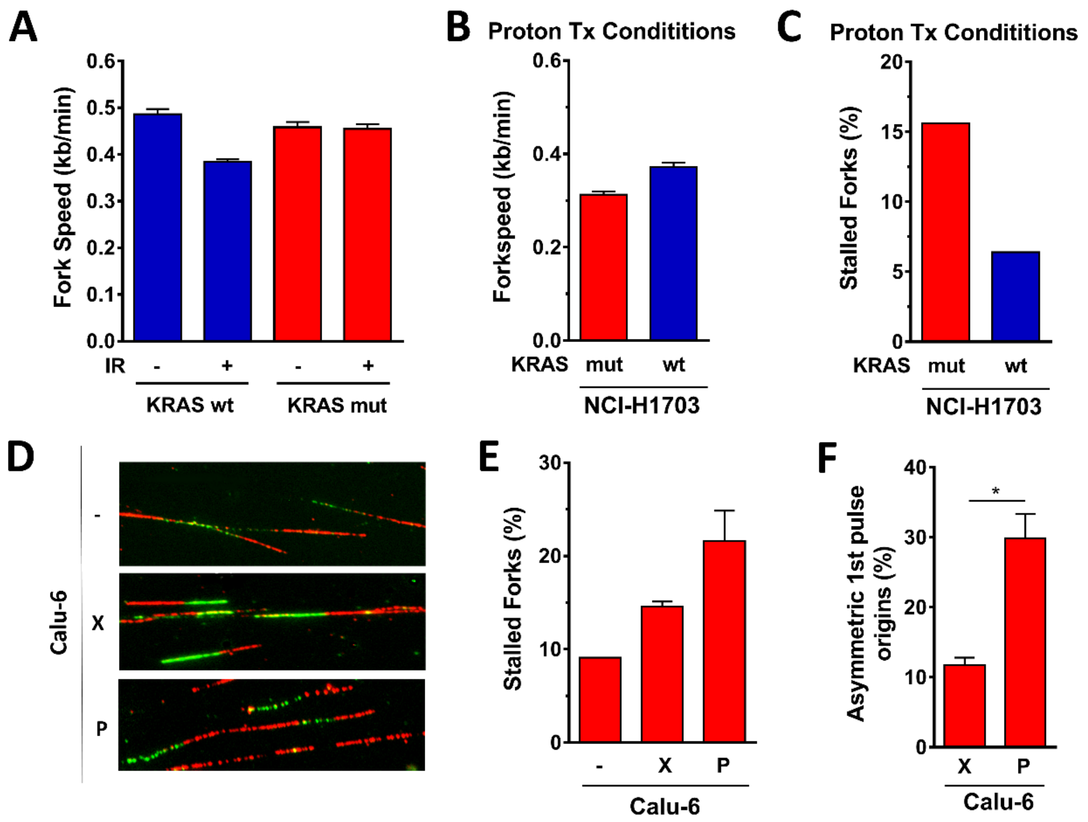

Supplementary Figure S4. Effects of X-ray and proton treatments. (**A**) Velocity of fork progression following ionizing radiation (IR) treatment (X-rays, 2 Gy) delivered in between the two pulse labels. (**B, C**) Assessment of velocity of fork progression and stalled forks with cells kept on ice for 2 hours in between first and second labeling to simulate proton treatment (Tx) conditions for which cells were transported to the Proton Center and back. (**D**) Representative images showing altered fork progression in proton (P) and X-ray (X) treated Calu-6 cells. (**E, F**) Percentage of stalled forks and asymmetric first pulse origins. All bars represent mean +/- SEM based on 2-3 independent repeats. Statistical comparison by two-sided T-test indicating * $p \leq 0.05$.

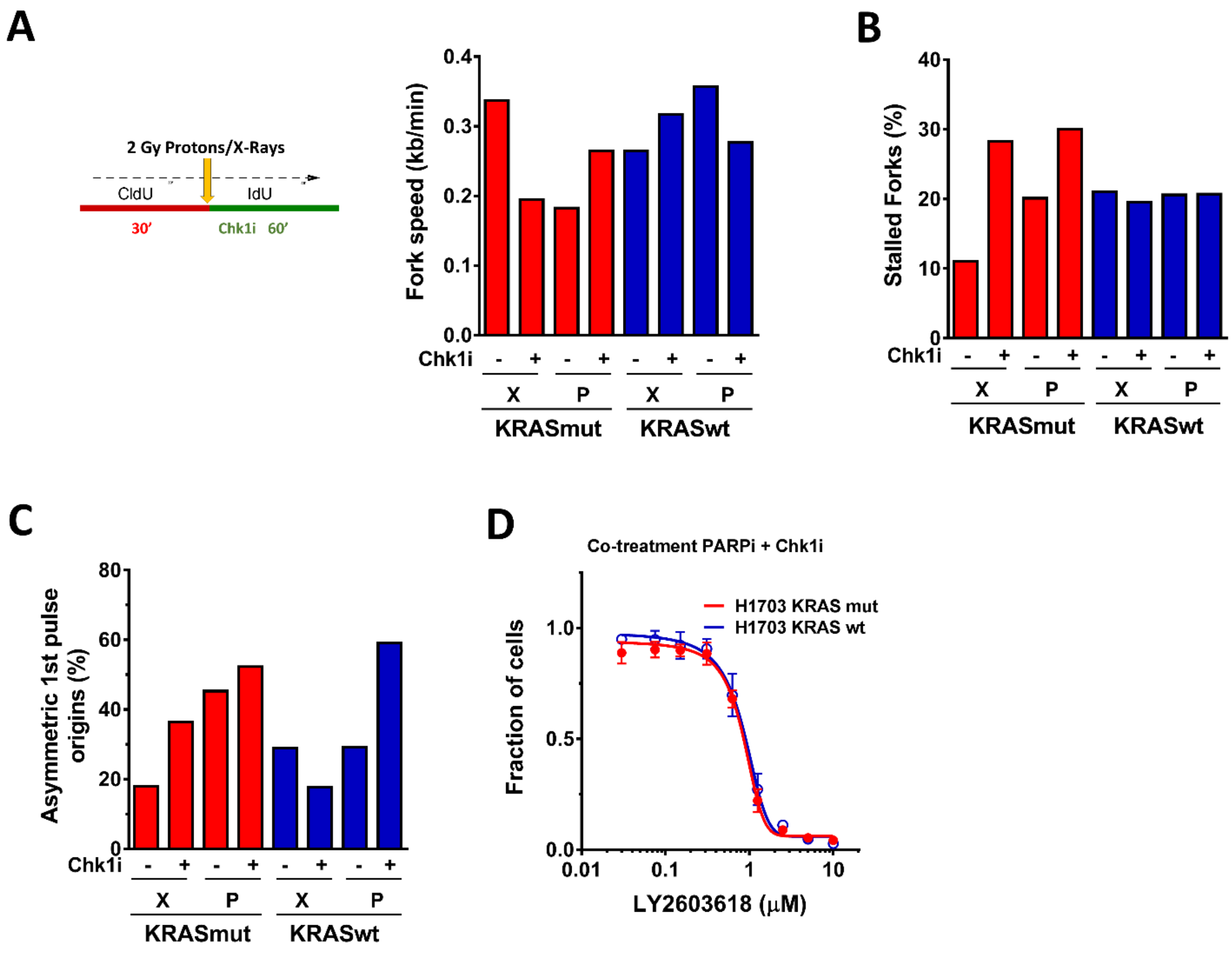

Supplementary Figure S5. Effects of Chk1 inhibition (Chk1i) on cellular responses to proton and PARP inhibitor treatments. (**A**) Left, Illustration of experimental setup for exploring effect of Chk1 inhibitor on response to proton (P) vs X-ray (X) treatments. Right, Velocity of fork progression following ionizing radiation (IR) treatment (X-rays, 2 Gy) delivered in between the two pulse labels. (**B**) Percentage of stalled replication forks. (**C**) Percentage of bidirectional asymmetric forks that progressed through replication fork arrest. (**D**) Fraction of NCI-H1703 cells after 5 days of combined olaparib (at IC70) and LY2603618 treatment.
